# Precise diagnosis and treatment of non-muscle invasive bladder cancer - A clinical perspective

**DOI:** 10.3389/fonc.2023.1042552

**Published:** 2023-01-31

**Authors:** Yongjun Yang, Chen Wang, Zonglin Li, Qiang Lu, Yuanwei Li

**Affiliations:** Department of Urology, Hunan Provincial People’s Hospital, The First Affiliated Hospital of Hunan Normal University, Changsha, Hunan, China

**Keywords:** bladder cancer, non-muscle invasive bladder cancer, precision medicine, en bloc resection, enhanced imaging, proteogenomics

## Abstract

According to the guidelines, transurethral resection of bladder tumor (TURBT) followed by intravesical therapy remains the standard strategy for the management of non-muscle invasive bladder cancer (NMIBC). However, even if patients receive standard strategy, the risk of postoperative recurrence and progression is high. From the clinical perspective, the standard strategy needs to be optimized and improved. Compared to conventional TURBT, the technique of en bloc resection of bladder tumor (ERBT) removes the tumor tissue in one piece, thus following the principles of cancer surgery. Meanwhile, the integrity and spatial orientation of tumor tissue is protected during the operation, which is helpful for pathologists to make accurate histopathological analysis. Then, urologists can make a postoperative individualized treatment plan based on the patient’s clinical characteristics and histopathological results. To date, there is no strong evidence that NMIBC patients treated with ERBT achieve better oncological prognosis, which indicates that ERBT alone does not yet improve patient outcomes. With the development of enhanced imaging technology and proteogenomics technology, en bloc resection combined with these technologies will make it possible to achieve precise diagnosis and treatment of bladder cancer. In this review, the authors analyze the current existing shortcomings of en bloc resection and points out its future direction, in order to promote continuous optimization of the management strategy of bladder cancer.

## Introduction

According to the latest cancer statistics, the incidence of bladder cancer (BCa) ranks tenth among malignant tumor diseases. In 2020, there will be approximately 573,000 new cases of BCa and 213,000 deaths, making it the second most common urological malignancy after prostate cancer (1). About 70% of newly diagnosed BCa present as early-stage (Tis, Ta, and T1) lesions confined to the mucosa or submucosa, termed non-muscle invasive bladder cancer (NMIBC) (2). Relevant guidelines recommend that the standard management strategy for NMIBC is transurethral resection of bladder tumor (TURBT) combined with postoperative individual intravesical chemotherapy or Bacillus Calmette-Guérin (BCG) immunotherapy based on risk stratification (3–5). TURBT is the first and most critical step in the diagnosis and treatment of NMIBC, as complete initial resection is essential for accurate risk stratification and good prognosis (6). Immediate postoperative intravesical instillation can kill exfoliated tumor cells and residual tumor tissue to reduce tumor recurrence (7).

One purpose of TURBT is to remove all visible tumor lesions in the bladder. During surgery, urologists mainly rely on indirect visual feedback and clinical experience to judge the depth and boundary of tumor invasion, but this subjective judgment is often inaccurate. A systematic review of clinical data from 8409 patients with high-grade Ta or T1 BCa showed that 17%-67% of Ta and 20%-71% of T1 patients were found to have residual tumors at the time of repeated transurethral resection (reTUR), and most of the residual tumors (36%-86%) were located at the original resection site (8). Due to the high incidence of residual tumors after initial resection, reTUR is recommended for patients diagnosed with primary T1 BCa and incomplete resection within 2-6 weeks (2). Previous studies or meta-analyses have shown that patients who have received reTUR benefit in terms of treatment, diagnosis and prognosis (9–11). A recent observational study based on a population of 7666 T1 BCa patients explored the association between reTUR and oncological prognosis. The results showed that reTUR is absolutely necessary for patients without detrusor samples after the initial resection, and that reTUR is likely to be beneficial to all patients diagnosed with T1 BCa regardless of the detrusor samples (12). The other purpose of TURBT is to obtain tumor samples for histopathological analysis. During conventional TURBT, piecemeal resection of tumor tissue obviously violates the principle of oncologic surgery (13). Meanwhile, the use of “incise and scatter” technique leads to thermal damage of tumor samples and fragmentation of tumor tissue, which makes accurate histopathological analysis of fragmented specimens difficult (14).

To overcome the drawbacks of conventional resection, en bloc resection of bladder tumor (ERBT) has emerged and has received increasing public attention over the past decade. Unlike piece-by-piece resection performed during TURBT, ERBT removes the tumor tissue in one piece, thereby alleviating the amount of exfoliated cancer cells that are suspected of causing out-of-field recurrence and migrating into the circulatory system (15). Unfortunately, to date, there is no strong clinical evidence that ERBT is superior to conventional resection in oncological outcomes (16). From a recent review on the recurrence mechanisms of NMIBC, undetected tumor tissue upon cystoscopy, local residual disease after initial resection and exfoliated cancer cells re-implantation might cause early disease recurrence, whereas drop metastasis from upper tract urothelial carcinoma and field change cancerization effects could lead to late disease recurrence (17). Therefore, we speculate that compared with conventional TURBT, ERBT cannot achieve a better prognosis for NMIBC patients by addressing some possible recurrence mechanisms, and this common aim of reducing the recurrence rate can only be achieved by tackling all possible recurrence mechanisms in a comprehensive manner. With the application and development of enhanced imaging technology and proteogenomics technology in BCa, en bloc resection combined with these technologies may tackle all possible recurrence mechanisms and achieve precise diagnosis and treatment of NMIBC.

## The technique of ERBT

Briefly, the surgical procedure of ERBT is to make a circular incision on the mucosa surrounding the tumor, and then perform blunt en bloc resection of the whole tumor, including detrusor muscle (18). Due to its potential advantages, ERBT has increased in popularity over recent years, especially in Europe and Asia. First, ERBT is considered to improve the quality of tumor specimens that allowing pathologists to better evaluate margin status for completeness of resection and depth of invasion (19). Second, the more controlled and refined en bloc resection may reduce the risk of perioperative complications such as obturator nerve reflex and bladder perforation (20). Third, the reduction in tumor fracturing leads to fewer floating tumor cells in the bladder, theoretically reducing the risk of tumor re-implantation and improving recurrence-free survival (RFS) rates (15, 21).

The main purpose of ERBT is to improve the quality of transurethral resection. It is believed that high-quality tumor resection can reduce the frequency of reTUR and the risk of recurrence. A surrogate for surgical quality is the presence of detrusor muscle (DM) within the tumor specimens (14). Compared to conventional TURBT, ERBT was associated with a higher percentage of DM present in histopathological specimens (22). The muscularis mucosa (MM) is composed of discontinuous smooth muscle bundles in the submucosa of the bladder wall, and the depth of tumor cell infiltration in the MM can be used for tumor T1a/b/c substaging. The recognition rate of MM was increased in specimens after ERBT compared with TURBT (23). A retrospective study used ERBT samples to analyze the correlation between T1 substaging and prognosis. The results showed that ERBT provided high-quality tumor specimens for MM recognition, and the prognosis of T1c BCa patients was poor (24). Pathologists were assigned to perform pT1 substaging on the TURBT and ERBT specimens, and the diagnostic time and diagnostic concordance rate of these pathologists were assessed. Compared with the use of TURBT samples, the use of ERBT samples shortened the diagnosis time of T1 substaging, and the diagnostic concordance rate was significantly better (25).

Compared with conventional TURBT, ERBT has its own advantages, but there are some limitations in the successful implementation of ERBT in reality. The major limitation in performing ERBT is the size of the tumor (26). In clinical practice, when stratified by tumor size, the technical success rates of ERBT for bladder tumors ≤3 cm and > 3 cm in size were 84.3% and 29.6%, respectively. Multivariate analysis showed that tumor size was the only significant factor predicting the success of ERBT surgery (27). Thus, in order to ensure a complete resection, urologists should accept modified approaches of ERBT, such as retrieval of tumor sample in several pieces (28–30), or piecemeal resection of the exophytic part of tumor tissue followed by en bloc resection of the tumor base (31). Meanwhile, several researchers have employed new devices to retrieve large tumor specimens. BCa with diameter ≤45 mm can be retrieved with Collins loop and laparoscopic forceps (32), and the tumor with diameter ≤75 mm can be retrieved with endo-bag commonly used in gastroenterology (33). The Chinese University of Hong Kong is currently conducting an exploratory study to investigate the feasibility of modified ERBT in patients with BCa more than 3 cm in diameter (ClinicalTrials.gov Identifier: NCT04081246) (34).

Like conventional TURBT, urologists can only rely on indirect visual feedback and clinical experience to judge the depth and boundary of tumor invasion during ERBT. En bloc re-resection was performed after the initial ERBT, and 6.41% of patients was found to have residual tumors (35). The detection rate of residual tumors during reTUR after ERBT and TURBT were 7% and 27.7%, respectively. There was no difference in RFS between the two groups during the follow-up period, but most tumor recurrence after ERBT occurred outside the original resection site (36, 37). The reason why RFS was not improved after ERBT may be partly because there was no difference between the two groups in identifying micro tumors during surgery, especially carcinoma *in situ* (CIS) (38). A systematic review was recently conducted to observe the results of random bladder biopsies in more than 10000 NIMBC patients, and the overall incidence of concurrent CIS was 17.35% (39). Furthermore, the rate would be reach up to 50% in patients with high-risk or sessile tumors (39, 40). Retrospective analysis showed that multifocality and T1 stage were independent prognostic factors for postoperative recurrence in high-risk NMIBC patients who underwent en bloc resection (41).

## Cooperate with pathologist to make accurate histopathological analysis

Pathological stage and histological grade, concurrent CIS, lymphovascular invasion (LVI) and histological variation play an important role in risk stratification of NIMBC (42). Tumor stage and grade, tumor status (primary or recurrent), previous intravesical chemotherapy, tumor size (<3cm vs. 3cm), and tumor focality (single vs. multiple) were often used as indicators of the scoring models to predict tumor recurrence (43). According to the International Collaboration on Cancer Reporting guidelines, apart from the pathological stage and histological grade, the pathological report of BCa specimens obtained by biopsy or transurethral resection should include the status of muscularis propria, histological variation, LVI and T1 substaging (44). Therefore, for the tumor specimens collected after ERBT, pathologists should focus on pathological stage, histological variation, LVI and related auxiliary techniques, such as immunohistochemistry, to evaluate these characteristics (45, 46). Meanwhile, the whole specimen section should be considered in the histopathological analysis to determine the status of the tumor margins (47).

The architecture and spatial orientation of tumor specimens were well preserved during en bloc resection, which was helpful for histopathological assessment. Close cooperation and comprehensive information sharing between urologists and pathologists are advocated to evaluate the clinicopathological characteristics of BCa and make an accurate risk stratification, and then formulate an optimal management strategy for patients with NMIBC. Despite ERBT failed to improve the recurrence rate, the more accurate histopathological analysis is likely to improve clinical decision-making and care delivery (48). T1 substaging provides important prognostic information on patients with primary high-risk NIMBC treated with BCG. Patients with extensive invasion of MM have a higher risk of BCG failure than patients with micro invasion. High-risk T1 substaging BCa has the potential to guide treatment decisions with BCG and alternative strategies at diagnosis (49). LVI is an important step in the cell dissemination of BCa, so it must be reported in histopathological analysis of biopsy and TURBT specimens. A meta-analysis including 65 studies found that LVI was positively correlated with disease recurrence and cancer-specific mortality regardless of pathological stage and node status (50). Integration of LVI status into predictive models might aid clinical decision-making regarding intravesical instillation regimens and schedules, early radical cystectomy (RC) in patients with high-risk T1 BCa and perioperative chemotherapy (51).

Whole slide image (WSI) system converts histopathological slides into digital high-resolution images with magnifications similar to conventional microscopy. WSI provides the possibility to create a digital three-dimensional (3D) histopathological reconstruction of BCa based on stacks of two-dimensional WSIs. 3D reconstructions show the added value of tumor morphology and may improve insight into the architectural changes and refine the risk stratification of BCa by providing detailed spatial and structural information (52). Meanwhile, 3D reconstruction can effectively identify the states of horizontal and vertical margins. pT1 residue was seen only in cases with positive vertical margins at the time of reTUR, whereas in patients with positive horizontal margins, pTa/pTis residues were seen at the original surgical site. Positive horizontal margins were not associated with RFS, but requires careful evaluation of residual tumors. A reTUR should be considered in patients with pT1 BCa with positive horizontal and vertical margins (53). En bloc resection provides sufficient specimens for tumor molecular pathology detection. Over the past few years, major insights have been gained into the molecular changes that occur during BCa development, but no consensus has been reached on how to incorporate these data into daily practice (54). Continuous multidisciplinary cooperation among urologists, pathologists, and oncologists have led to standards in the pathological reporting and microscopic diagnosis of BCa specimens. Emerging tumor molecular insights already affect the understanding and reporting of BCa by healthcare workers, and are likely to have a greater impact with increasing data and standardization of analysis.

## ERBT combined with enhanced imaging technology

Performing ERBT under white light cystoscopy (WLC) may ignore the small or occult malignant lesions, especially CIS. Surgical margin status affects prognosis and reTUR outcomes. Patients with negative tumor margins of pT1 BCa can omit reTUR after ERBT, while patients with positive horizontal or vertical margins should be recommended for reTUR until proven otherwise (55). CIS is a high-risk NMIBC confined to the mucosa, which is easily confused with inflammatory lesions due to its similar structural appearance (56). The presence of DM in tumor specimens, the absence of concomitant CIS and en bloc resection were able to predict a negative histology at reTUR, which opens the door to avoid reTUR in a very carefully selected patient cohort (57).

As an auxiliary means of WLC, new optical enhanced imaging technologies such as photodynamic diagnosis (PDD) and narrow-band imaging (NBI) have shown the potential to improve the detection rate of BCa and highlight the tumor boundary (58, 59). Compared with WLC-assisted TURBT, PDD or NBI-assisted TURBT can improve the sensitivity of intraoperative diagnosis and reduce the postoperative recurrence rate (60). The results of a recent network meta-analysis support the combination of PDD-assisted TURBT and concomitant single immediate intravesical chemotherapy as the optimal initial treatment for patients with NMIBC (61). Even with high-quality TURBT, when PDD is routinely used for transurethral resection, PDD-assisted TURBT seems to be associated with a significantly lower recurrence rate after 3 years of “real-life” experience, especially in high-risk patients (62). Photosensitizers can promote the generation of reactive oxygen species inside cells under specific wavelength irradiation, and then play the role of photodynamic therapy (PDT). Studies have shown that the combination of PDD-assisted TURBT and PDT is an effective and safe option for the first-line treatment for NMIBC (63, 64). Compared with PDD-assisted TURBT, adequate histopathological assessment of muscle invasion was significantly better in postoperative tumor specimens after PDD-assisted ERBT, suggesting its clinical potential to reduce the rate of early reTUR (65).

Real-time intraoperative guidance is essential for complete and safe tumor resection during transurethral resection. The rapid development of fluorophores with excellent physicochemical characteristics, detection instruments and targeting strategies led to the first clinical trials of targeted near-infrared (NIR) fluorophores for intraoperative tumor detection in the early 2010s (66). Fluorescence-guided imaging shows the potential to guide surgeons during complex surgical interventions, which can provide real-time guidance for surgeons to delineate tumor boundary and help surgeons find residual diseases invisible to the naked eye or computed tomography (67–69). CD47, a member of the immunoglobulin superfamily, is overexpressed in more than 80% of bladder tumor cell membranes, but not in normal urothelium (70). Our previous studies showed that CD47 antibody conjugated to a NIR fluorophore can be used for fluorescence imaging of BCa (71, 72). CD44 is a cancer stem cell marker, which is highly expressed on the cell membrane of BCa and participates in cell adhesion, cell migration, tumor progression and metastasis (73). The results of the first human experiment showed that the fluorescence-guided imaging based on the NIR fluorescence probe targeting CD44v6 is a safe and effective method, which can improve the detection rate of BCa, and may enable complete resection to prevent recurrence (74). The pH-low insertion peptide (pHLIP) is a C-helix derived from the bacteriorhodopsin protein that targets the acidic microenvironment in the tumor matrix (75). pHLIP conjugated to a NIR fluorophore can be used for ex vivo fluorescence imaging of BCa. The results showed that the sensitivity and specificity of tumor lesions detection were 97% and 100%, respectively (76). Therefore, en bloc resection assisted by the new optical enhanced imaging technology draws on the advantages of both, and is expected to improve the quality and completeness of transurethral resection.

## ERBT combined with proteogenomics technology

At a median follow-up of 3-8 years, 21% of high-risk NMIBC developed disease progression, and the overall survival rate after disease progression was 35% (77). The current management standard for T1 BCa consists of induction and maintenance of intravesical BCG or early RC. Guidelines recommend intensive postoperative follow-up with cystoscopy and cytology, and periodic cross-sectional imaging (3–5). In recent years, genomic and transcriptomic analysis have perfected precision medicine in oncology care. Molecular classification of high-risk T1 BCa may improve the poor RFS and progression-free survival by adjusting surveillance schedules and escalating discussions regarding RC. Due to the inherent complexity and interaction of tumor genetics, the tumor immune microenvironment and the responsiveness of host immune factors to BCG treatment, no biomarker of T1 BCa has been identified so far (78). Therefore, there is an urgent need to find biomarkers that distinguish different T1 molecular subtypes (79).

Next generation sequencing of untreated NMIBC demonstrated that the majority of NMIBC had at least one potentially actionable alteration that could serve as a target in rationally designed trials of intravesical or systemic therapy. DNA damage response and repair gene alterations were frequent in high-grade NMIBC and were associated with increased mutational burden, which may have therapeutic implications for BCG immunotherapy and immune checkpoint inhibitor therapy. ARID1A mutations were associated with an increased risk of recurrence after BCG therapy. For such patients, close follow-up, immune checkpoint inhibitor therapy or early RC can be recommended (80). Integrative multi-omics analysis was performed on patients diagnosed with NMIBC, and transcriptomic analysis identified four categories (1, 2a, 2b and 3) that reflect tumor biology and disease aggressiveness. High chromosomal instability, p53-pathway disruption and APOBEC-related mutations were significantly associated with transcriptomic class 2a and inferior oncological prognosis (81). To better understand the molecular heterogeneity of T1 BCa, transcriptome profiling and unsupervised clustering were performed and five consensus subtypes of T1 tumors were identified. T1-Myc and T1-Early subtypes had the highest median MYC expression rate, and RFS was significantly lower than the other three subtypes (82).

## Future perspectives

BCa is a common malignant tumor of the genitourinary system and one of the tumors with the highest treatment cost (83). High-grade NMIBC is a heterogeneous disease, and treatment modalities include intravesical maintenance BCG and RC. Intravesical maintenance BCG therapy can preserve the bladder, with little change in health-related quality of life during treatment, but there is a risk of delaying treatment due to disease progression. As such, urologists should inform patients about the relative risks of each treatment modality and recommend the use of an individualized risk-adaptive approach (84). In principle, scientists fully agree with the prediction that molecular classification of BCa will become the “gold standard” of precision medicine in the future. However, given the reality of the currently available genomic data on BCa (most of the work is done in muscle invasive bladder cancer, but very little in NMIBC) and the utility of clinically available patient-specific data, there is an urgent need for further exploration of genomic data from NMIBC patients (85).

**Figure 1:**
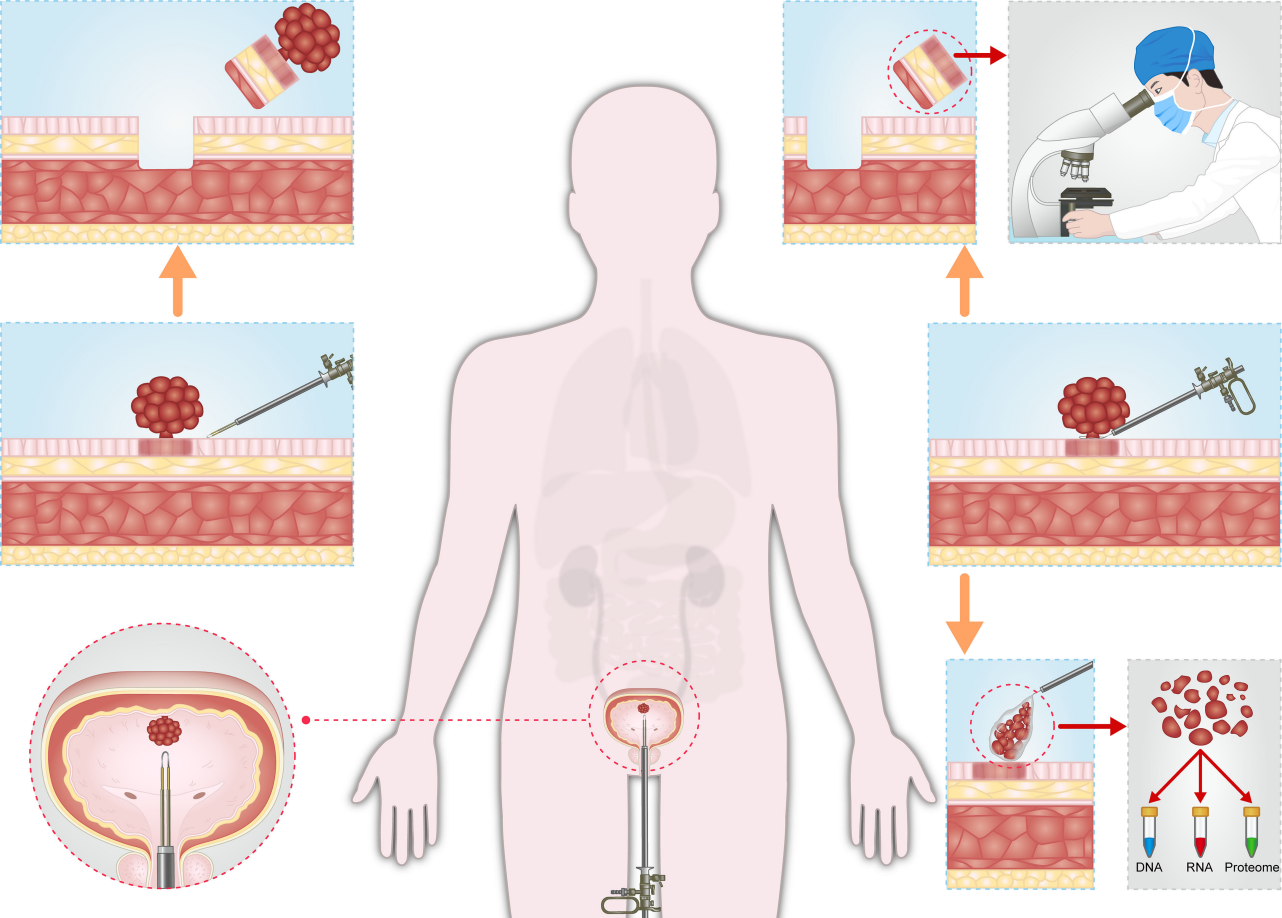
Schematic diagram of the precise diagnosis and treatment of NMIBC. (**Left**). For patients with suspected NMIBC, when the tumor diameter is less than 3cm, the whole tumor tissue is resected under the guidance of enhanced optical imaging. The urologist can completely remove tumor tissue under the guidance of enhanced optical imaging, and the pathologist can make accurate histopathological analysis after obtaining tumor samples. Complete transurethral tumor resection and accurate tumor risk stratification are beneficial to reducing the risk of recurrence and progression after surgery. (**Right**). When the tumor was larger than 3 cm in diameter, the exophytic part of the tumor was first removed in a whole piece, collected in an elastic bag and crushed into several pieces, and then taken out from the bladder for proteogenomics analysis. The tumor base containing DM was then en bloc resected for histopathological analysis. The whole tumor exophytic part collected in the elastic bag provides sufficient pathological specimens for proteogenomics analysis, while the tumor base containing DM facilitates histopathological analysis by pathologists, including T1 substaging, LVI, and histological variation.

For patients with suspected NMIBC, when the tumor diameter is less than 3cm, the whole tumor tissue is resected under the guidance of enhanced optical imaging. The urologist can completely remove tumor tissue under the guidance of enhanced optical imaging, and the pathologist can make accurate histopathological analysis after obtaining tumor samples. Complete transurethral tumor resection and accurate tumor risk stratification are beneficial to reducing the risk of recurrence and progression after surgery. When the tumor was larger than 3 cm in diameter or those with solid appearance, multiparametric MRI (mpMRI) should be considered before operation to determine the depth of tumor invasion (86). For the tumor tissue with no sign of detrusor invasion on mpMRI, the exophytic part of the tumor was first removed in a whole piece, collected in an elastic bag and crushed into several pieces, and then taken out from the bladder for proteogenomics analysis. The tumor base containing DM was then en bloc resected for histopathological analysis. The whole tumor exophytic part collected in the elastic bag provides sufficient pathological specimens for proteogenomics analysis, while the tumor base containing DM facilitates histopathological analysis by pathologists, including T1 substaging, LVI, and histological variation (**Figure 1**).

Overall, as an adjunct to WLC, the new optical enhanced imaging technology has shown the potential to improve the detection rate of BCa and highlight the tumor boundary, leading to high-quality and completeness of transurethral tumor resection. Proteogenomics analysis of tumor specimens provides urologists with insight into molecular classification and therapeutic targets, leading to successful clinical translation. Our hope is that ERBT combined with this technologies may provide precise diagnosis and treatment for patients with NMIBC.

## Author contributions

YY wrote the first draft of the manuscript. CW, ZL, QL, and YL edited sections of the manuscript. All authors contributed to the article and approved the submitted version.
